# Multiscale image denoising using goodness-of-fit test based on EDF statistics

**DOI:** 10.1371/journal.pone.0216197

**Published:** 2019-05-10

**Authors:** Khuram Naveed, Bisma Shaukat, Shoaib Ehsan, Klaus D. Mcdonald-Maier, Naveed ur Rehman

**Affiliations:** 1 Department of Electrical and Computer Engineering, COMSATS University Islamabad (CUI), Islamabad, Pakistan; 2 School of Computer Science and Electronic Engineering, University of Essex, Colchester, United Kingdom; New York Institute of Technology, UNITED STATES

## Abstract

Two novel image denoising algorithms are proposed which employ goodness of fit (GoF) test at multiple image scales. Proposed methods operate by employing the GoF tests locally on the wavelet coefficients of a noisy image obtained via discrete wavelet transform (DWT) and the dual tree complex wavelet transform (DT-CWT) respectively. We next formulate image denoising as a binary hypothesis testing problem with the *null hypothesis* indicating the presence of noise and the *alternate hypothesis* representing the presence of desired signal only. The decision that a given wavelet coefficient corresponds to the *null hypothesis* or the *alternate hypothesis* involves the GoF testing based on empirical distribution function (EDF), applied locally on the noisy wavelet coefficients. The performance of the proposed methods is validated by comparing them against the state of the art image denoising methods.

## 1 Introduction

The acquisition and transmission normally corrupt an image by introducing an additive noise. In this regard, image denoising algorithms are utilized to suppress noise while preserving the desired image features. Let *x*_*p*,*q*_ denote a pixel of a noisy *N* × *N* sized image **X** at location (*p*, *q*), acquired from an acquisition device, a transmission medium or a reconstruction process as

$$\begin{array}{c} x_{p,q}=s_{p,q}+\eta_{p,q}, \end{array}$$ (1)

where *s*_*p*,*q*_ denotes the pixels of the true image **S** while *η*_*p*,*q*_ denotes noise at pixel location (*p*, *q*). In matrix form, the above equation can be written as

$$\begin{array}{c} X=S+\eta . \end{array}$$ (2)

The goal of denoising is to estimate the true signal **S** from its noisy observation **X**. Here, ***η*** is considered an independent Gaussian noise $N(0,\sigma^{2})$ with zero mean and arbitrary variance *σ*^2^.

Earlier, denoising was achieved by linear methods such as Weiner filtering in the Fourier domain [1]. However, the scope of such techniques is only limited to stationary data because the Fourier transform is incapable of handling non-linear or non-stationary data. That resulted in multi-scale denoising methods employing non-linear operations such as thresholding in the transform domain [2]. For that purpose, discrete wavelet transform (DWT) was employed which decomposes a dataset into multiple scales that gives a sparse representation of the signal in transform domain [3]. The DWT based denoising algorithms exploit the sparsity of the wavelet coefficients [4–6] through simple yet powerful nonlinear thresholding operations [7, 8] to obtain the denoised image. Similar principle is adopted while denoising with variants of the DWT like double density discrete wavelet transform (DDDWT), complex wavelet transform (CWT), dual tree complex wavelet transform (DT-CWT) etc.

Among the wavelet based denoising methods, *VisuShrink* [9] is one of the simplest techniques; it employs a universal threshold for all the scales depending largely on image size and noise level. The disadvantage of this method is that it tends to over smooth large sized images. This is due to the dependence of the estimated threshold on the input image size. Therefore, comparatively better performance is shown by the adaptive data driven techniques which estimate the threshold separately for each scale [10–18]. An example of such a method is the *SureShrink* [10], which exploits the Stein’s unbiased risk estimator (SURE) to get an unbiased estimate of the threshold to perform signal/image denoising. An extension of the *SureShrink* is the *Surelet* [12], which employs the principle of SURE along with the linear expansion techniques (LET) to cast the denoising problem as the one with linear system of equations. The *BayesShrink* [13], on the other hand, operates within the Bayesian framework with prior application of Generalized Gaussian Distribution (GGD) on wavelet coefficients. An empirical Bayes approach of denoising based on the Jeffrey’s non-informative prior [14] exploits the sparsity and de-correlation properties of DWT for denoising purposes. Recently, empirical Bayes approach of denoising has been extended to 2D scale-mixing complex valued wavelet transform, namely *cSM-EB* [15].

Sparsity based signal recovery methods have also been explored as an avenue for image denoising. To that end, a compressive sensing based image denoising algorithm is proposed in [19] where *L*_1_-minimization has been used to recover the true signal. In [20], sparse and redundant signal representation over learned dictionaries is used for denoising images. Clustering based locally learned dictionaries are employed for image denoising in [21] whereby clusters of local patches are obtained based on likewise geometrical structures. Similarly, clustering based sparse representation (CSR) method for image denoising combines the dictionary learning with structured clustering to exploit enhanced sparsity in [22]. A hybrid image denoising algorithm is proposed in [23] based on wavelet transform in combination with the learned and redundant dictionaries. In this method, the wavelet transform is used to obtain multiscale feature and sparse prior for wavelet coefficients which leads to the sparse representation in wavelet domain. Subsequently, the *K-SVD* algorithm is used to build sparse over-complete dictionaries of wavelet coefficients resulting in a state of the art image denoising algorithm. Patch based noisy image specific orthogonal dictionaries are learned using PCA in [24] to threshold the patch coefficients for image denoising, namely *PaPCA*.

A collaborative hard thresholding based filtering technique is used within BM3D [25] to exploit enhanced sparsity of transform domain. Here, a complex multistage process is adopted starting with the grouping of similar fragments of 2D transformed coefficients which are then arranged into 3D data arrays. Subsequently, attenuation of noise is achieved via spatial collaborative hard-thresholding followed by the collaborative Weiner filtering on the 3D arrays of the transformed coefficients. Despite its efficacy, the computational complexity of BM3D is considerably large owing to its complicated multi-step procedure [25].

Sparsity driven iterative algorithms are also used to solve total variation (TV) minimization for image denoising. For instance, several iterative algorithms have been designed for TV denoising including iterative soft thresholding algorithm (ISTA), fast ISTA (FISTA) and a monotone version of FISTA [26]. In addition, split Bregman algorithm has been used for efficient isotropic and anisotropic TV image denosing in [27]. Similarly, Beltrami regularization is considered in [28] for image denoising and has been shown to outperform TV based methods.

Spatial domain filtering techniques such as mean and median filtering are commonly used but are known to produce sub-optimal denoising. However, an efficient spatial domain non local mean (NLM) filtering technique for image denoising is proposed in [29], which happens to be a gold standard denoising method owing to its effective denoising performance. In this technique, image pixels having smallest euclidean distance from each other are grouped together leading to weighted mean of these pixels for noise smoothing. Hence, for each pixel, similar pixels are searched, grouped and averaged leading to very high computational complexity. Though, this technique yields visually pleasing denoising results but it is known to over-smooth details of an image.

Mostly, classical thresholding strategies exploit sparsity in transform domain by considering that coefficients corresponding to the signal have higher amplitudes compared to the noisy coefficients. Contrarily, Cai and Silverman [16] observed that wavelet coefficients corresponding to signal are distributed in the locality of each other while coefficients corresponding to noise are distributed uniformly. They used this fact to introduce neighbourhood based thresholding strategies for 1D signals [16] in which a coefficient is classified as signal if it is surrounded by likewise coefficients and vice versa. *NeighShrink* [17] introduces neighbourhood based thresholding to image denoising which operates by classifying a wavelet coefficient surrounded by higher amplitude coefficients as desired signal while a coefficient surrounded by the lower amplitude coefficients is classified as noise. Similarly, *NeighSure* [18] refines neighbourhood based thresholding via the SURE to achieve image denoising. A simple yet effective image denoising method exploiting the statistical neighbourhood dependencies of wavelet coefficients is proposed in [30]. A statistical model for neighbourhoods of oriented pyramid coefficients is developed in [31], which is based on Gaussian scale mixtures of empirical wavelet coefficients. The intra-scale dependencies within the wavelet coefficients have been modeled using fuzzy features in *Fuzzy-Shrink* [32], where a fuzzy feature distinguishes between the image discontinuities and noise.

Recently, statistical methods have emerged as a strong tool in the wavelet based image denoising. These methods exploit statistical dependencies within the wavelet coefficients for estimating the thresholds for denoising. *BiShrink* [33] models inter-scale dependencies in wavelet coefficients (obtained via the DWT as well as the DT-CWT) based on a new non-Gaussian bivariate distribution for threshold estimation. The method also includes a nonlinear bivariate shrinkage function driven through a maximum *a posteriori* (MAP) estimator. The *ProbShrink* [32] estimates a threshold based on the probability that a given coefficient contains significant information (signal of interest) by assuming a generalized Laplacian prior for noise free data.

A major issue in the conventional DWT is the lack of translation invariance in the traditional wavelet basis functions resulting in artifacts in the aftermath of denoising. These artifacts could be explained by the Gibbs phenomena in the neighbourhood of discontinuities. Stationary DWT, which is rotation invariant, can render partial translation invariance to the denoising results and can be implemented via *cycle spinning* approach [34]. In *cycle spinning*, noisy data is first shifted left or right, denoised via a wavelet based method and subsequently un-shifted. This process is repeated several times and all the results are averaged to produce a denoised signal/image with lesser artifacts. It has been shown in [34] that denoising results can be improved considerably by making the DWT partially translation invariant through cycle spinning.

In contrast to DWT, the DT-CWT enjoys near translation invariance and directional selectivity at the cost of a higher degree of redundancy [35]. The redundancy in DT-CWT is due to the fact that real and imaginary parts of the complex wavelet coefficients are dealt as independent wavelet coefficients which makes it twice redundant. However, in order to incorporate directional selectivity in the two dimensional DT-CWT, the complex wavelet coefficients are obtained at six directions compared to the three directions of the DWT (i.e. horizontal, vertical and diagonal), which further increases the redundancy by two. Hence, the two dimensional DT-CWT is 4:1 redundant as compared to the DWT [35]. In the two dimensional DT-CWT, dual tree of filters oriented at 6 directions are employed, yielding six bands of real parts and six bands of imaginary parts of the complex wavelet coefficients at each scale.

The directional selectivity in DT-CWT preserves orientation of the edges or discontinuities having a line or a curve shape, unlike DWT which only preserves the point discontinuities. In addition, the directional selectivity in DT-CWT helps avoid the checker-board artifacts during denoising process by differentiating between the edges oriented at 45° and −45° [35].

The redundancy, in combination with the filter banks designed to achieve complex number representation, makes DT-CWT approximately translation invariant. The maximal decimation in DWT causes aliasing in the decomposed wavelet coefficients. In order to cancel the effect of aliasing and achieve perfect reconstruction, the synthesis filters for inverse DWT operation are designed to fulfill the aliasing-free condition. However, the aliasing can only be avoided if the wavelet coefficients are not perturbed, which is not the case in wavelet based denoising. Contrarily, in DT-CWT, the inherent redundancy (4:1) suppresses aliasing to a large extent, yielding better denoising results.

Several denoising methods have been reported in literature which utilize the above desirable properties of the DT-CWT: In [30], dependencies among three scales of DT-CWT coefficients are exploited. *NeighSure* [18] employs Stein’s unbiased risk estimator (SURE) on complex wavelet coefficients of the DT-CWT to find an optimum threshold and a window size. Furthermore, image denoising methods reported in [36–41] are some of the recent methods which exploit near translation invariance and directional selectivity of the DT-CWT for improved denoising performance.

In this paper, two image denoising methods are proposed which employ statistical goodness of fit (GoF) tests on multi-scale wavelet coefficients obtained via DWT and DT-CWT. The decision process regarding the presence of noise at multiple scales is based on the statistical GoF tests, wherein Anderson Darling (AD) statistic is used as a measure of similarity between the local wavelet coefficients and reference Gaussian noise distribution. A coefficient is detected as corresponding to noise if its associated AD measure is less than a threshold, which is a function of probability of false alarm. Those coefficients are then eliminated (set to zero) while the remaining coefficients are retained. We demonstrate the effectiveness of the proposed methods by comparing them against the state-of-the-art in wavelet based image denoising on both natural and medical input images.

In our previous work [42–45], we had employed GoF test on multiple 1D signal scales, obtained via the 1D DWT, for signal denoising. Also, Poisson denoising in the context of CMOS/CCD images has also been proposed in [46]. In this work, we employ GoF test on multiple image scales for image denoising. To this end, a novel framework is developed for GoF testing on multiple scales of DWT as well as the DT-CWT, which offers better translation invariance and directional selectivity. The proposed methodology is significantly different from classic wavelet thresholding techniques in which the wavelet coefficients are directly compared against a threshold. In the proposed thresholding method, decision regarding the noisy image coefficients is made based on the statistical distance between the distribution or model of the local wavelet coefficients from the reference noise distribution.

This paper is organized as follows: Section II gives the background of wavelet based image denoising along with an insight into the GoF testing and its operation. A detailed discussion on the proposed algorithms is presented in Section III. Section IV presents the experimental results and discussion, while Section V concludes the paper while also highlighting possible avenues for future work.

## 2 Theoretical background

### 2.1 Wavelet transform based image denoising

**Fig 1:**
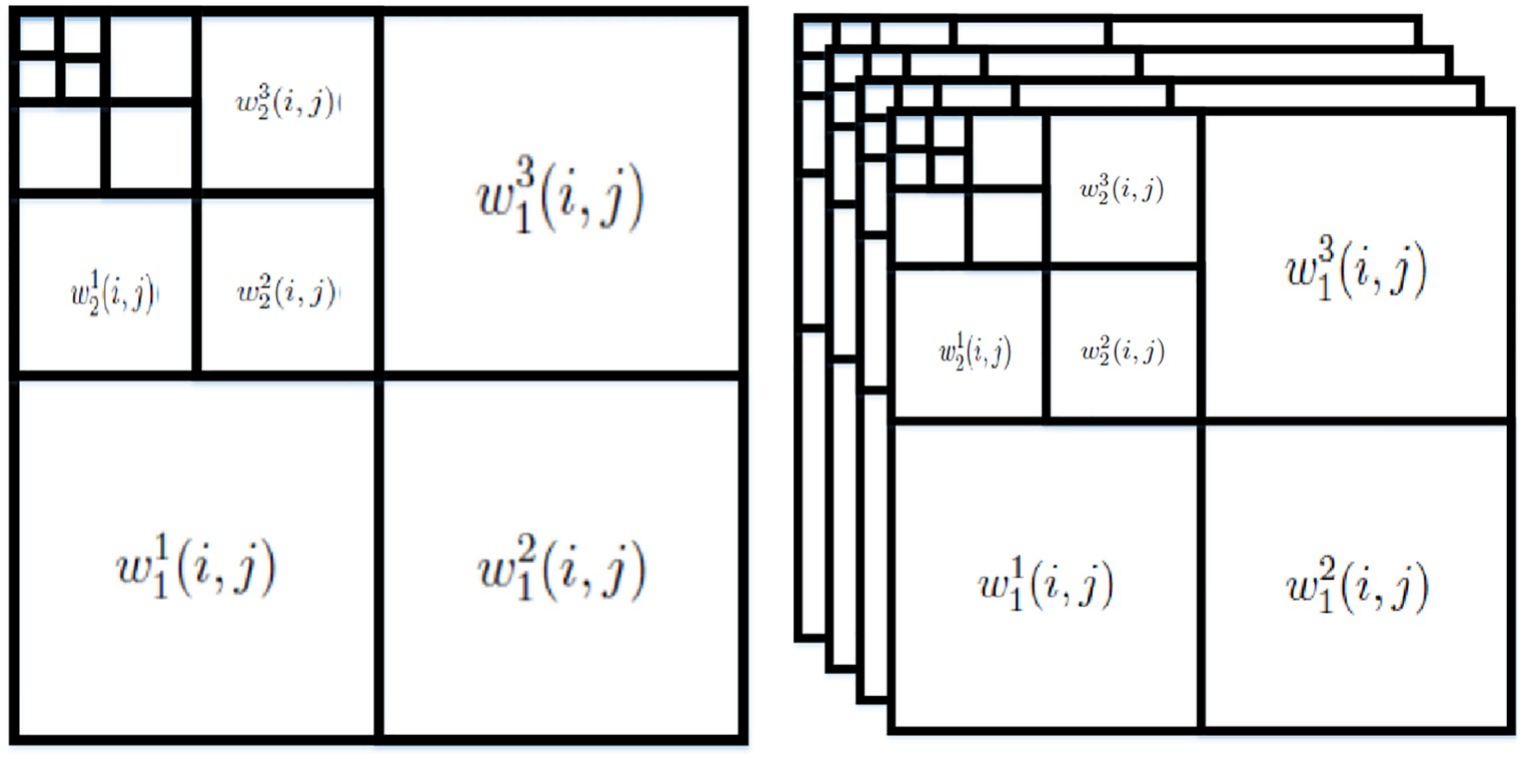
Difference in the formation of wavelet coefficient matrix W in case of the DWT and the DT-CWT operation; (left) arrangement of the empirical wavelet coefficients in a 2D matrix W in case of the DWT operation; (right) arrangement of the complex wavelet coefficients in a 3D matrix W in case of the DT-CWT operation, where first two layers contain the real parts and the last two layers contain the imaginary parts of the complex wavelet coefficients.

Let W denote the wavelet transform operated over a noisy image **X** to decompose it into wavelet coefficients at multiple scales as

$$\begin{array}{c} W=W(X), \end{array}$$ (3)

where **W** denotes the matrix composed of wavelet coefficients $w_{i}^{j}$ with *j* denoting the scale of decomposition, *i* denotes location of a coefficient at multiple scales. The operator W may refer to the DWT or the DT-CWT operation: when W refers to DWT, **W** is a two dimensional matrix of wavelet coefficients $w_{i}^{j}$ and its formation is depicted in Fig 1 (left), where each scale of decomposition contains three bands of wavelet coefficients, each of which is associated to a direction namely horizontal, vertical and diagonal. The location index *i* first lists the horizontal coefficients (column wise) followed by the listing of vertical and diagonal wavelet coefficients.

On the other hand, when the operator W denotes the DT-CWT operation, **W** is a three dimensional matrix of wavelet coefficients as shown in Fig 1 (right), where each scale of decomposition contains twelve bands of wavelet coefficients. In order to achieve this representation we placed the redundant wavelet coefficients, yielded via DT-CWT, in four different two dimensional matrices in accordance with the formation shown in Fig 1 (left) and then those four matrices are placed above each other to make four layers of a three dimensional matrix as shown in Fig 1 (right). It must be noted that first two layers contain the real parts of the complex wavelet coefficients and last two layers contain the imaginary parts of the complex wavelet coefficients for each scale).

A threshold value *T* is next estimated to classify the coefficients as belonging to signal or noise i.e. a popular universal threshold $T=\sigma \sqrt{2log(N\times N)}$ [9] is based on image size *N* × *N* and noise standard deviation *σ* which is estimated as

$$\begin{array}{c} \sigma =\frac{median(|\left\{w_{1}^{i}\;\forall \;i∼\;\text{diagnal}\;\text{coefficients}\right\}|}{0.6745}, \end{array}$$ (4)

here *i* denotes the index of only the diagonal wavelet coefficients at the scale *j* = 1. A thresholding operator *ϒ* is next applied individually on each wavelet coefficient as given below

$$\begin{array}{c} {\hat{w}}_{i}^{j}=ϒ\left(w_{i}^{j}\right), \end{array}$$ (5)

where ${\hat{w}}_{i}^{j}$ are thresholded empirical wavelet coefficients, *ϒ* could be soft or hard thresholding rule which exhibit near optimal properties in minimax sense and better convergence rates for approximating functions in Besov spaces [7, 8]. In the soft thresholding operation, the signal elements less than threshold *T* are floored to zero and the amplitudes of the remaining signal elements are reduced (shrunk) by *T*. The hard thresholding operation keeps the signal elements whose values are greater than *T* and sets the remaining coefficients to zero.

After performing thresholding operation, inverse wavelet transform [3] is applied on the noise suppressed wavelet coefficients to get an estimate $\hat{S}$ of the true image **S** in the spatial domain

$$\begin{array}{c} \hat{S}=W^{-1}\left\{\hat{W}\right\}, \end{array}$$ (6)

where $\hat{W}$ are thresholded empirical wavelet coefficients ${\hat{w}}_{i}^{j}$ (see Fig 1).

### 2.2 Statistical goodness-of-fit testing

The goodness-of-fit (GoF) test indicates how well a specified model or distribution fits a given set of observations. The GoF test performs hypothesis testing whereby the case with observations or data fitting the specified model/distribution is termed as *null hypothesis* $H_{0}$ and the case where observation reject the specified model/distribution is termed as alternate hypothesis $H_{1}$. In order to quantify the difference between the observed values and the values expected under the specified distribution, different statistics/measures of GoF have been defined [47, 48]. Several measures of GoF test are employed in practice [49–52], each having unique properties of their own but only the Anderson Darlington (AD) statistics [51] will be discussed here because of its relevance with our work. A detailed discussion on the topic is presented in [53].

Let $F\left(t\right)=\sum_{t}1\left(z>t\right)$ denote the empirical cumulative distribution function (ECDF) of input samples **z** with support *t* and $F_{r}\left(t\right)=\int_{t}p\left(z>t\right)dz$ represent the hypothesized cumulative distribution function (reference CDF) corresponding to a probability density function *p*(*z*). The AD statistic *τ* is given as follows

$$\begin{array}{c} \tau =\int_{-\infty }^{\infty }{\left(F_{r}\left(t\right)-F\left(t\right)\right)}^{2}\psi \left(F_{r}\left(t\right)\right)d\left(F_{r}\left(t\right)\right), \end{array}$$ (7)

where $\psi (F_{r}\left(t\right))$ is the weighting function responsible for giving more weight to the tail of the distribution function $F_{r}\left(t\right)$ is given as

$$\begin{array}{c} \psi \left(F_{r}\left(t\right)\right)=(F_{r}\left(t\right)\left(1-F_{r}\left(t\right)\right){)}^{-1}. \end{array}$$ (8)

In order to compute *τ*, numeric expression for the AD statistic relation in (7) is as follows

$$\begin{array}{c} \tau =-L-H, \end{array}$$ (9)

where *L* denotes the size of the given observations *x*_*t*_ or the size of window in case of local operation of GoF test and *H* is defined as

$$\begin{array}{c} H=\sum_{t=1}^{L}\frac{\left(2t-1\right)}{L}(\text{ln}\left(F_{r}\left(z_{t}\right)-\text{ln}\left(F_{r}\left(z_{L+1-t}\right)\right)\right). \end{array}$$ (10)

The probability distribution of distance *τ* is specified asymptotically as window lengths *L* → ∞.

Within the framework of GoF test, a threshold *T* is computed for error probability of given observations falsely reject the reference distribution. In spectrum sensing related literature [54–56], the probability of falsely rejecting a candidate distribution is termed as the probability of false alarm *P*_*fa*_, defined as follows,

$$\begin{array}{c} P_{fa}=Prob\left\{\tau >T|H_{0}\right\}=\int_{\left\{z\;s.t.\;\tau >\lambda \right\}}p\left(z|H_{0}\right)dz \end{array}$$ (11)

where the range {*z s*.*t*. *τ* > λ} are the values yielding false alarm. *P*_*fa*_ is generally kept very very low to estimate an appropriate threshold *T* [57].

Next, hypothesis testing defined in (15) is performed to validate the null hypothesis $H_{0}$ or reject it i.e. the alternate hypothesis $H_{1}$.

$$\begin{array}{c} \begin{array}{cc} & H_{0}:\tau \leq T;\\ & H_{1}:\tau >T. \end{array} \end{array}$$ (12)

## 3 GoF based multiscale image denoising

Two novel image denoising methods are proposed which employ GoF test on the wavelet coefficients of the noisy image obtained by using DWT and DTCWT respectively. The DT-CWT exhibits approximate translation invariance and directional selectivity which helps it to suppress the artifacts otherwise present in the DWT based denoising results. We denote the proposed denoising methods as the *GoFShrink* based on the DWT and the DT-CWT.

Conventionally, GoF tests have been applied to detection problems where they operate directly on input data to test the binary hypothesis of *noise only* and *signal plus noise* cases e.g. spectrum sensing [54–56], as follows

$$\begin{array}{c} \begin{array}{cc} & H_{0}:x\in \text{noise},\\ & H_{1}:x\in \text{signal}\;\text{+}\;\text{noise}. \end{array} \end{array}$$ (13)

Contrarily, in the *denoising problem*, the alternate hypothesis $H_{1}$ must correspond to the detection of *signal only* case. To achieve that, we propose to employ multiscale wavelet transforms on the input noisy data before applying the GoF test. The DWT and DT-CWT distribute the signal coefficients sparsely as compared to noise coefficients which are distributed uniformly across the scales, thus segregating signal and noise into separate coefficients at multiple scales. The *modified binary hypothesis* using the GoF test at multiple scales are given bellow

$$\begin{array}{c} \begin{array}{cc} & H_{0}^{{}^{\prime }}:w_{i}^{j}\in \text{noise},\\ & H_{1}^{{}^{\prime }}:w_{i}^{j}\in \text{signal}, \end{array} \end{array}$$ (14)

where $H_{0}^{{}^{\prime }}$ and $H_{1}^{{}^{\prime }}$ denote modified null and alternate hypothesis at multiple scales respectively and $w_{i}^{j}$ denotes multiscale wavelet coefficients obtained through DWT or the DT-CWT operation as specified in (3).

Given a scale dependent threshold *T*_*j*_, the proposed framework first computes a test statistic *τ*_*i*_ for a sub-image centered around the coefficient $w_{i}^{j}$ at scale *j* and then compares it with the threshold *T*_*j*_. The decision regarding the null hypothesis $H_{0}^{{}^{\prime }}$ or alternate hypothesis $H_{1}^{{}^{\prime }}$, as defined in (14) is taken as follows

$$\begin{array}{c} \begin{array}{cc} & H_{0}^{{}^{\prime }}:\tau_{i}\leq T_{j};\;\;\;i.e.\;\;\;w_{i}^{j}\in \text{noise},\\ & H_{1}^{{}^{\prime }}:\tau_{i}>T_{j};\;\;\;i.e.\;\;\;w_{i}^{j}\in \text{signal}. \end{array} \end{array}$$ (15)

**Fig 2:**
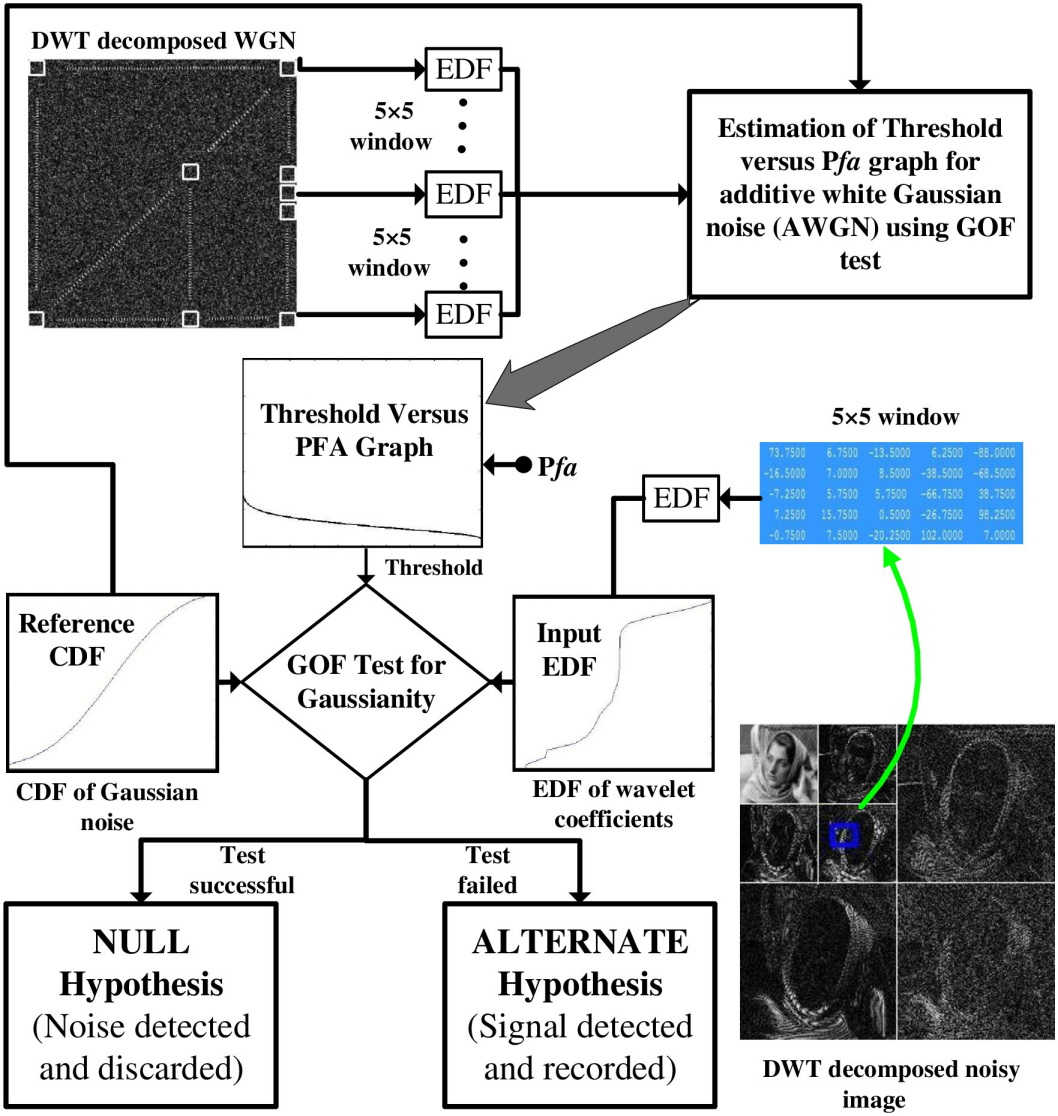
Block diagram of the *GoFShrink* based on DWT.

Finally, the coefficients identified as noise (i.e. $H_{0}^{{}^{\prime }}$) samples are rejected at each scale, while the remaining coefficients are retained as part of the desired signal (i.e. $H_{1}^{{}^{\prime }}$). The steps of the proposed algorithm are listed in the Algorithm 1 and are graphically depicted in Fig 2.

**Remark 1**: For the GoF testing, the reference CDF $F_{r}\left(t\right)$ (i.e. CDF describing noise in the signal) must be known a-priori. In our case, the reference distribution is white Gaussian noise which means specifying mean and variance completely specifies *F*_*r*_(*t*).

**Fig 3:**
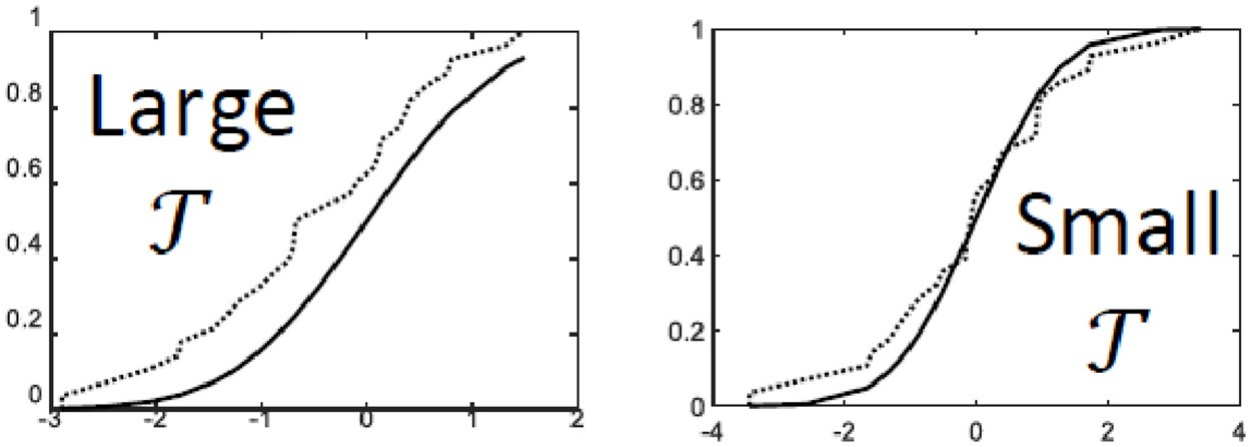
Test for Gaussianity via GoF tests where the case (a) shows noise detection as *τ* is expected to small; and the case (b) shows signal detection as *τ* is expected to large.

**Remark 2**: *τ* could be computed using any GoF based empirical distribution function (EDF) statistic e.g. Anderson Darling (AD), Cramer Von Mises (CVM) and Kolmogrov Smirnov (KS) statistics etc. AD and CVM have been found to be relatively robust as compared to other EDF statistics. An insight into how these statistics ensure detection of signal only and noise only cases, is shown in Fig 3.

Let an input noisy image **X** be decomposed into wavelet coefficients **W** at multiple scales *j* = 1 ‥ *J* through the DWT operation W in (1). We next estimate the standard deviation of noise *σ* in the input image via (4) and subsequently normalize the wavelet coefficients by the *σ* to make the noise unit variance at multiple scales, as follows,

$$\begin{array}{c} \tilde{W}=\frac{W}{\hat{\sigma }}. \end{array}$$ (16)

where $\tilde{W}$ denotes the normalized DWT coefficients.

Next, the level dependent threshold *T*_*j*_ must be computed for a probability of false alarm *P*_*fa*_ which requires the estimation of $F_{r}\left(t\right)$; the reference noise distribution at scale *k*. In this work, the reference distribution at multiple scales corresponds to zero mean white Gaussian noise i.e., $N(0,\sigma^{2})$ since DWT and DT-CWT retain the Gaussianity of input noise at multiple scales and can be computed as follows,

$$\begin{array}{c} F_{r}\left(t\right)=\int_{-\infty }^{t}\frac{1}{\sqrt{2\pi \sigma }}\;e^{\frac{z^{2}}{\sigma^{2}}}dz \end{array}$$ (17)

where *z* is a zero mean Gaussian random variable with arbitrary variance *σ*^2^ which can be estimated using (4). The EDF $F^{i}\left(t\right)$ of local wavelet coefficients around the coefficient $w_{i}^{j}$ at scale *j* is computed as

$$\begin{array}{c} F^{i}\left(t\right)=\sum_{t=1}^{l\times l}1.\left(w_{i}^{j}>t\right), \end{array}$$ (18)

where *l* × *l* denote the window size.

**Fig 4:**
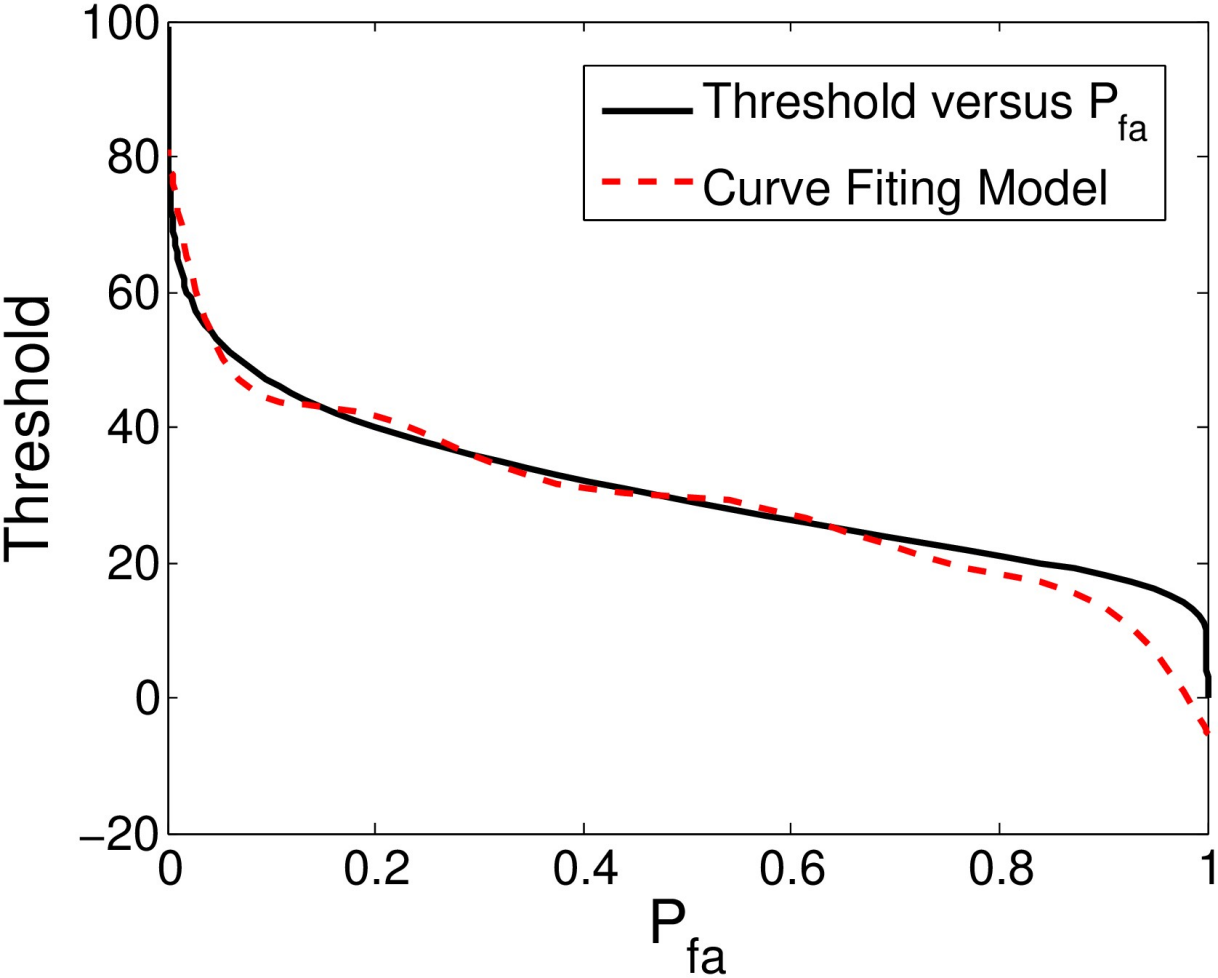
Threshold versus *P*_*fa*_ graph generated empirically for the first five scales of wavelet decomposed Gaussian noise along with its curve fitted version.

For empirically estimating *T*_*j*_ at scale *j*, a large sized WGN ***η*** is decomposed using the DWT and the resulting multiscale WGN coefficients **W**_*η*_ are divided into small windows of size *l* × *l*. Let $L_{j}$ be the total number of such windows at scale *j*. For each window centered at *i*, let *τ*_*i*_ be the value of AD statistic computed via (7) by employing the $F_{r}\left(t\right)$ and $F^{i}\left(t\right)$ defined in (17) and (18) respectively. If *T*_*j*_ be a chosen threshold then let $M_{j}$ be the number of false alarms where *τ*_*i*_ ≥ *T*_*j*_, then the $P_{fa}\left(T_{j}\right)=\frac{M_{j}}{L_{j}}$. This way, the *P*_*fa*_ versus threshold curve is estimated for a range of values of threshold *T*_*j*_ as shown in Fig 4.

**Remark 3**: Owing to the orthogonal and linear nature of the DWT, the *T*_*j*_ versus *P*_*fa*_ curves were found to be similar for all the scales as expected. The following mathematical model for threshold selection based on *P*_*fa*_ was obtained using polynomial curve-fitting as shown in Fig 4.

$$\begin{array}{c} \begin{array}{cc} & T_{k}^{l}\left(p\right)=42950{\left(P_{fa}\right)}^{8}-193200{\left(P_{fa}\right)}^{7}+357600{\left(P_{fa}\right)}^{6}\\ & -351900{\left(P_{fa}\right)}^{5}+198400{\left(P_{fa}\right)}^{4}-64360{\left(P_{fa}\right)}^{3}\\ & +11470{\left(P_{fa}\right)}^{2}-1047\left(P_{fa}\right)+81.76. \end{array} \end{array}$$ (19)

**Remark 4**: Probability of false alarm (*P*_*fa*_), in this case, denotes the probability that a noise coefficient is detected as a signal. That probability should be very small and is specified in the range of *P*_*fa*_ = 10^−3^ → 10^−5^.

Let ${\tilde{w}}_{i}^{j}$ be the wavelet coefficients which are part of $\tilde{W}$, the GoF test is applied on each ${\tilde{w}}_{i}^{j}$ by taking a window of size *l* × *l* around ${\tilde{w}}_{i}^{j}$ and then computing their EDF $F^{i}\left(t\right)$ using (18). Subsequently, the AD distance *τ*_*i*_ between the $F^{i}\left(t\right)$ and the reference CDF $F_{r}\left(t\right)$ at scale *j* is estimated via (7). For a given *P*_*fa*_, a threshold *T*_*j*_ is selected and the following GoF based thresholding function is employed,

$$\begin{array}{c} {\hat{w}}_{i}^{j}=\{\begin{array}{cc} 0 & \;if\;\;\tau_{i}\;\;\leq T_{j}\\ {\tilde{w}}_{i}^{j} & \;if\;\;\tau_{i}>T_{j}. \end{array} \end{array}$$ (20)

**Fig 5:**
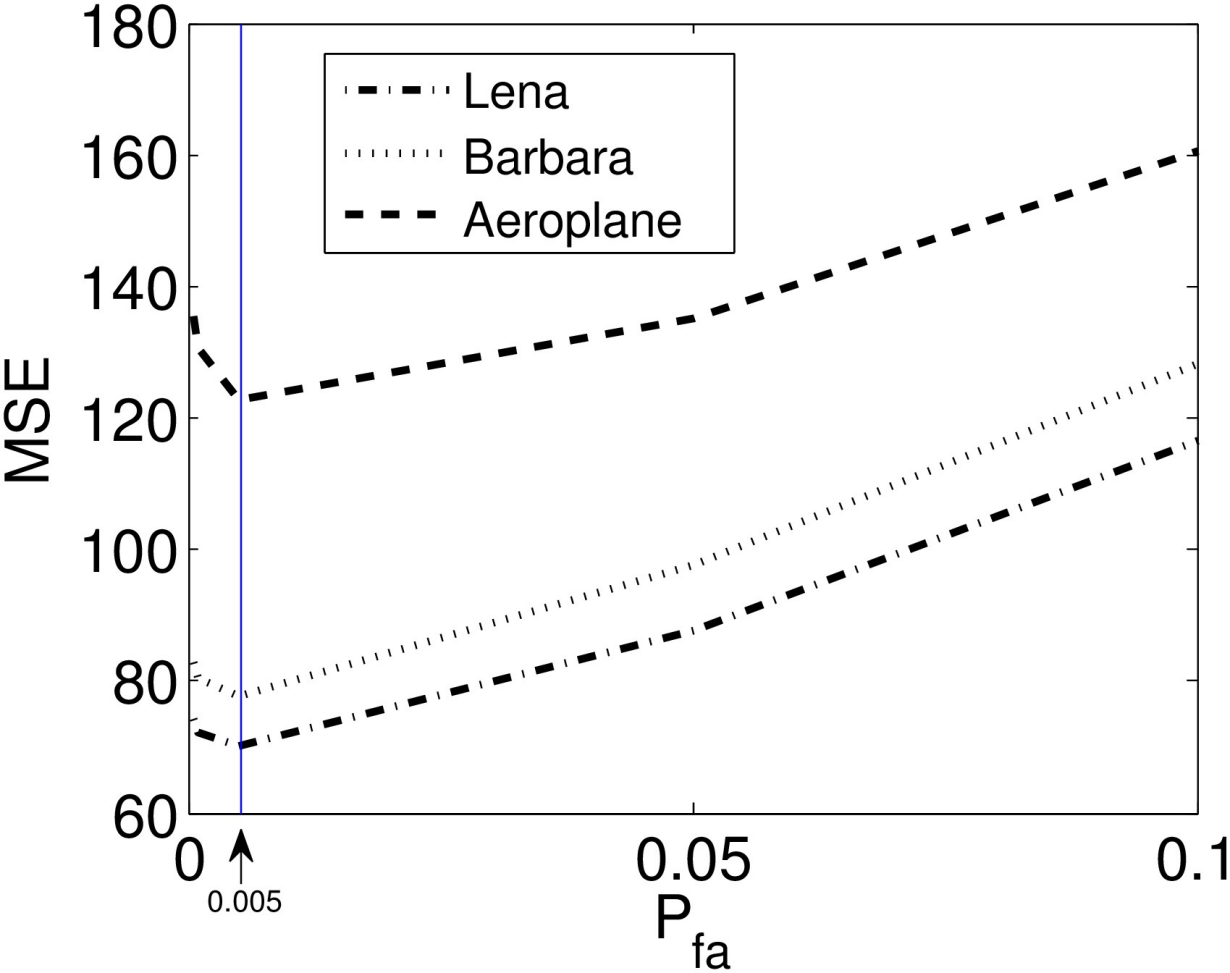
Empirical selection of *P*_*fa*_: Mean squared error (MSE) versus the *P*_*fa*_ relation obtained empirically for several test images. Notice that the *P*_*fa*_ values closer to zero yield better results.

Fig 5 reports an experimental estimation of a suitable choice of *P*_*fa*_ for selecting the thresholding *T*_*j*_.

**Remark 5**: The thresholding function (20) performs hard thresholding on the wavelet coefficients. This is in-line with the neighbourhood based thresholding rules reported in [16–18, 30, 31], whereby the central coefficient of a neighbourhood or a window is either retained as desired signal or removed as noise based on statistical or deterministic dependencies between the local wavelet coefficients.

Finally, the denoised empirical wavelet coefficients are reconstructed by inverse DWT operation to yield the estimate ${\hat{S}}_{p,q}$ of the true image **S**_*p*,*q*_. However, before the reconstruction, the normalization process in step 2 is reversed by multiplying all the retrieved signal coefficients with the estimated variance of the noise.

$$\begin{array}{c} \hat{S}=\left\{W^{-1}\left(\hat{\sigma }\times \hat{W}\right)\right\}. \end{array}$$ (21)

Subsequently, cycle spinning operation defined in [34] is performed to obtained denoised image. We shall denote the proposed algorithm by *GoFShrink-TI* in the remainder of this paper.

The above method can be extended to DT-CWT by applying the GoF test has been employed on the complex wavelet coefficients obtained by applying the DT-CWT on the noisy image. The DT-CWT exhibits near translation invariance and directional selectivity, which enables it to suppress various artifacts otherwise present in the DWT based denoising results [58].

**Fig 6:**
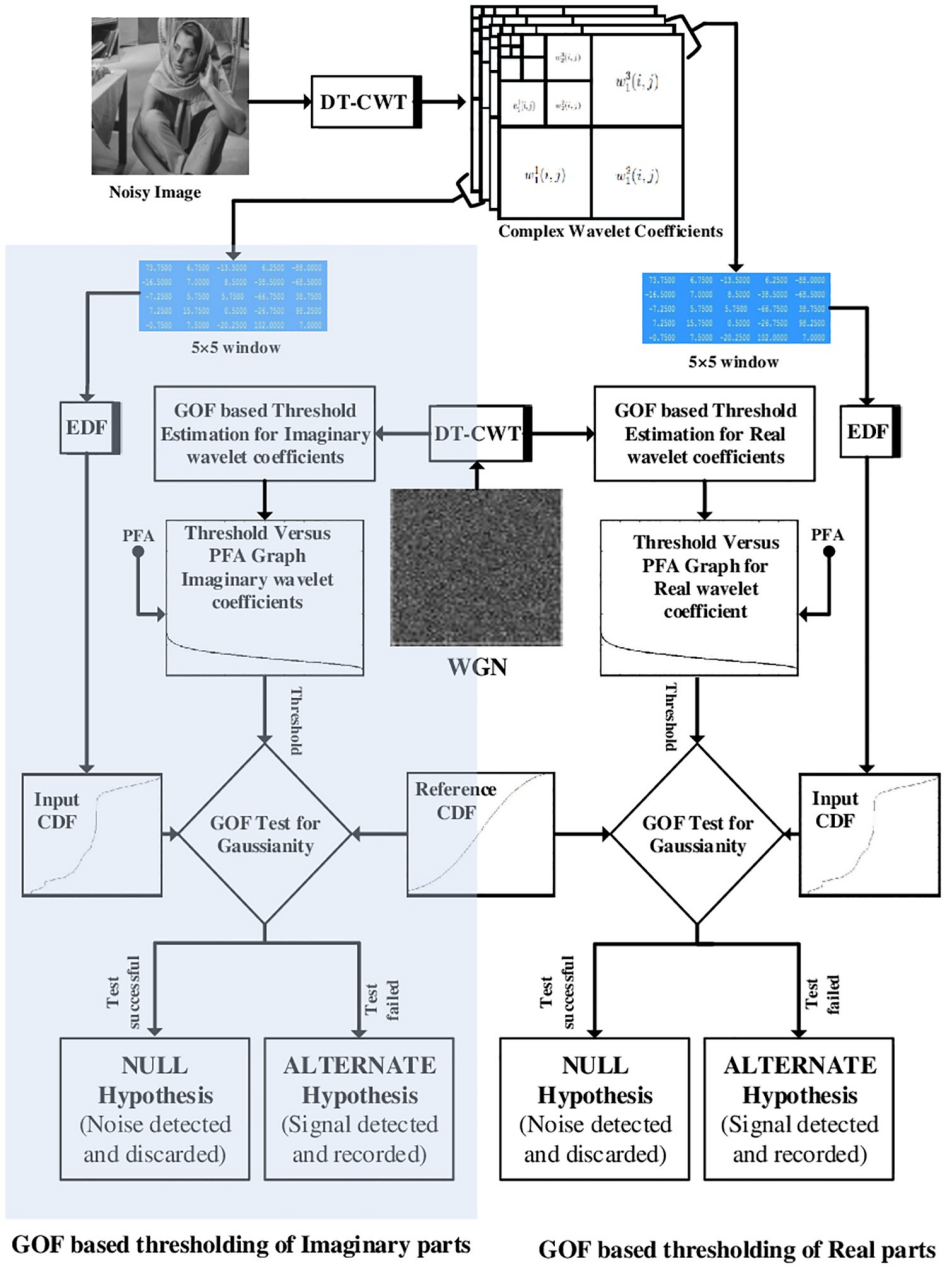
Block diagram of the *GoFShrink* based on DT-CWT.

The DT-CWT yields complex wavelet coefficients by separately calculating their real and imaginary parts. We propose to apply GoF based denoising operation, namely *GoFShrink*, separately on both sets of real and imaginary parts. These steps include: (i) calculation of the scale dependent thresholds for the real and imaginary trees of noisy wavelet coefficients (a graphical depiction of this process is shown in Fig 6 (middle)); (ii) computation of the complex wavelet coefficients **W** of the noisy image by employing (1), where W denotes the DT-CWT operation; (iii) normalization of the DT-CWT coefficients of the noisy signal by employing (16); (iv) performing the GoF based thresholding in parallel, whereby AD statistics was employed independently on the real and imaginary DT-CWT coefficients locally, followed by the use of thresholding function in (20) for detecting and annihilating coefficients belonging to noise while the remaining coefficients are retained as desired signal (the shaded region in Fig 6 shows this process for imaginary parts while the unshaded region shows the same for real parts); (v) taking the inverse-DT-CWT operation, after the reverse normalization operation, to yield the denoised signal. For the rest of the paper, we will denote this method by *GoFShrink-DT*. Matlab code of both of the proposed methods is available online at https://www.mathworks.com/matlabcentral/fileexchange/64531-gofshrink.

**Algorithm 1** GoFShrink based on DWT

1: *i*, *j* ← 0                  ⊳ 2D Wavelet coefficient indexes

2: W←W(X)               ⊳ DWT operation on input **X**

3: *P*_*fa*_ ← 0.005        ⊳ *P*_*fa*_ selection based on the experiment given in Fig 5

4: $T_{k}^{l}\leftarrow T\left(W_{\eta },P_{fa}^{\left(k,l\right)}\right)$  ⊳ Operation T implemented via the procedure given at Fig 2 (left)

5: $\hat{\sigma }\leftarrow \frac{median(|{\left\{w_{1}^{2}\left(i,j\right)\right\}}_{i,j=1,\;...\;,\frac{N}{2}}|)}{0.6745}$      ⊳ Noise variance estimation

6: $\tilde{W}\leftarrow \frac{W}{\hat{\sigma }}$            ⊳ Normalisation of the wavelet coefficient

7: **for** *k* = 1 to *K* **do**

8:  **for** *l* = 1 to 3 **do**

9:   **for** $i,j=1\;\text{to}\;\frac{N}{2^{k}}$ **do**

10:    $\tau_{k}^{l}\left(i,j\right)=-L\;-\sum_{n=1}^{L}\frac{\left(2n-1\right)}{L}(\ln\left(F_{r}\left({\tilde{w}}_{n}\right)\right)-\ln(F_{r}\left({\tilde{w}}_{L+1-n}\right))$    ⊳ AD statistic

11:    **if** $\tau_{k}^{l}\left(i,j\right)\leq T_{k}^{l}$ **then**

12:     ${\hat{w}}_{k}^{l}\left(i,j\right)\leftarrow 0$       ⊳ Noise detection during GoF test

13:    **else**

14:     ${\hat{w}}_{k}^{l}\left(i,j\right)\leftarrow {\tilde{w}}_{k}^{l}\left(i,j\right)$    ⊳ Signal detection during GoF test

15:    **end if**

16:   **end for**

17:  **end for**

18: **end for**

19: $\hat{S}\leftarrow W^{-1}\left(\hat{W}\times \hat{\sigma }\right)$                ⊳ Inverse DWT

## 4 Computational complexity

In this section we present the computational cost of the *GoFShrink based on DWT*. The computational cost of the *GoFShrink based on DT-CWT* will be four times to that of *GoFShrink based on DWT*, provided the length of filters used by both transforms is exactly the same.

The DWT operation on an image (of size *N* × *N*) involves separate filtering of the rows and columns, where first rows are processed via 1D low and high pass filters followed by the decimation by 2, and then the same process is applied on the columns of the input matrix.

If *M* denotes the size of the 1D low and high pass filters then the computation of the DWT coefficients will take 2*M* multiplications and 2(*M* − 1) additions per sample point. Since at *k*th level, the coefficients in the rows will be down sampled by 2^*k*−1^, the total cost of implementing a filter at *k*th level will involve 2*M*(1 − 2^−*k*^) multiplications and 2(*M* − 1)(1 − 2^−*k*^) additions per sample point. The total number of coefficients processed by row filters will be *N*^2^ as there are *N* rows in the image with each row having *N* number of pixels. Hence, the total complexity for implementing the row filters at all scales becomes 2*N*^2^ *M*(1 − 2^−*k*^) multiplications and 2*N*^2^(*M* − 1)(1 − 2^−*k*^) additions. After including the computational cost on image columns, which is the same as that on the rows, the total computational cost of the 2D DWT operation on the noisy image will be 4*N*^2^ *M*(1 − 2^−*k*^) multiplications and 4*N*^2^(*M* − 1)(1 − 2^−*k*^) additions. Next, these DWT coefficients will be normalized by the estimated noise standard deviation which required *N*^2^ multiplications.

The computation of the empirical CDF F(x) is an important part of GoF tests and will require the computations of the order of *O*(*LlogL*) where *L* denotes total number of coefficients in the $\sqrt{L}\times \sqrt{L}$ window which are to be used for the GoF test.

From (10), we can see that the computation of the AD statistics measure will require 3*N*^2^*L* multiplication and 2*L*(*L* − 1)*N*^2^ additions for the *N*^2^ coefficients of the DWT.

At the end, the inverse DWT operation will be performed on the thresholded wavelet coefficients. The inverse DWT operation mirrors the operation of the forward DWT but with different filters having the same length *M*. Therefore, the computational complexity of the inverse DWT will be exactly the same as the forward DWT operation.

## 5 Experimental results

This section presents the performance comparison of the proposed algorithms against the state of the art in image denoising. The peak signal to noise ratio (PSNR) has been employed as the measure of quantitative performance, given as

$$\begin{array}{c} \text{PSNR}=10\;{\text{log}}_{10}\;\left(\frac{255^{2}}{\text{MSE}}\right)\;\;dB. \end{array}$$ (22)

The mean squared error (MSE) is calculated as

$$\begin{array}{c} \text{MSE}=\frac{1}{N^{2}}\sum_{p=1}^{N}\sum_{q=1}^{N}\left(s_{p,q}-{\hat{s}}_{p,q}\right), \end{array}$$ (23)

where *s*_*p*,*q*_ denotes pixels of the true image **S** of size *N* × *N* and ${\hat{s}}_{p,q}$ represents the pixels of the denoised image $\hat{S}$. Note that MSE of noisy image is equal to the variance of the noise *σ*^2^.

For qualitative analysis, we employ the structural similarity (SSIM) measure and feature similarity (FSIM) measure. While SSIM evaluates the quality of a recovered image based on the structure, the FSIM evaluates the subjective quality of the recovered image based on how the human visual system (HVS) perceives the quality of an image [59].

**Fig 7:**
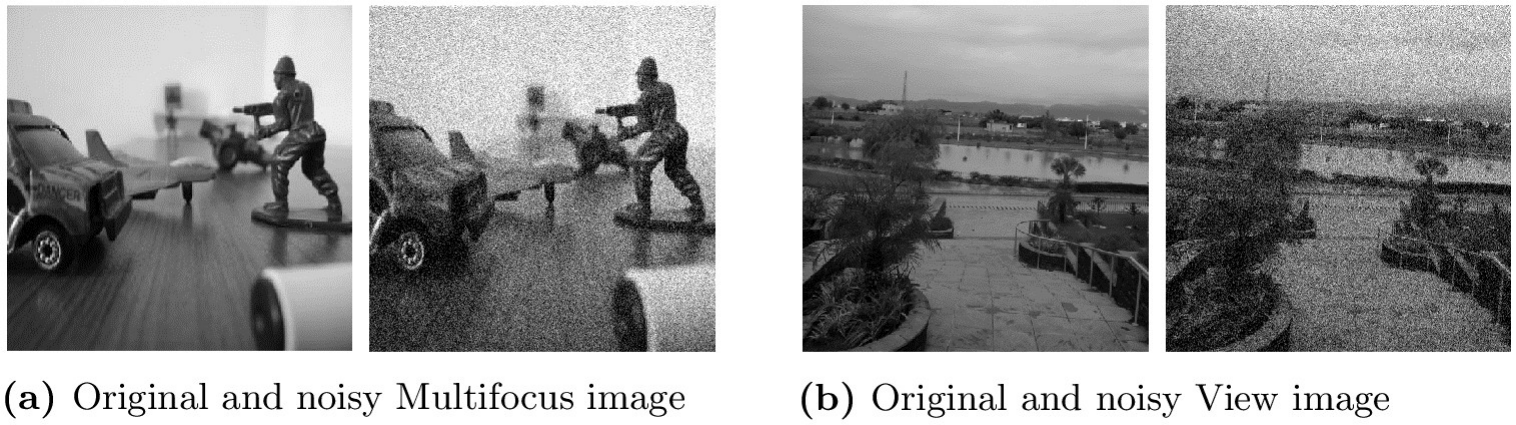
Selected input images along with their noisy versions at noise level *σ* = 30 namely, (a) Multi-focus image; (b) View image.

The set of input images used for experimentation consisted of standard test images including *Lena, Barbara, Peppers, Aeroplane* and *Cameraman* images coupled with images used in other practical applications such as medical *Brain MRI* image, a diffused *Multi-focus* image and a natural *View* image. The *Brain MRI* image was taken from the NIH IMAGE program ImageJ (https://imagej.nih.gov/nih-image/about.html), a public domain software package distributed freely by the National Institutes of Health. The *Multi-focus* image set was acquired during the study in [60]. The *View* image was selected due to higher amount of details in it and is captured by authors at COMSATS University Islamabad campus using a 13 mega-pixel digital camera. These test images were corrupted by Gaussian noise at multiple noise levels corresponding to *σ* = 10, 20, 30, 40 and 50, which produces noisy images with PSNRs = 28.13, 22.11, 18.59, 16.07 & 14.15 respectively. The *Multi-focus* image and *View* image are displayed in Fig 7 along with their noisy versions, while *Lena*, *Barbara*, *Peppers*, *Aeroplane*, *Cameraman* and *Brain MRI* have been provided as a supplementary material with this work in S1 Fig.

The performance of the proposed *GoFShrink-TI* and *GoFShrink-DT* methods have been evaluated by comparing them against the well known state of the art image denoising methods based on different variants of the wavelet transform: *BayesShrink (DWT)* [13], *BiShrink (DT-CWT)* [33], *Surelet (DWT)* [12], *NeighSure (DT-CWT)* [18], *cSM-EB (CWT)* [15]. In addition to the wavelet based methods, sparsity driven methods like *PaPCA* [24], *iTVD* [27], *aTVD* [27] and *BeltDen* [28] have also been considered for comparison. Computationally expensive technique *non local mean* (NLM) filtering method [29] has also been used as a comparative denoising method on practical images.

The DWT based denoising methods including the proposed *GoFShrink-TI* were implemented using *Daubechies* wavelet filters of eight taps, namely *db8*. The noisy images were decomposed into *D* = 5 wavelet levels. For the DT-CWT based image denoising methods, namely the *NeighSure*, *BiShrink*, and the proposed *GoFShrink-DT*, the dual tree of wavelet filters developed by Kingsbury in [61] for complex wavelets, were employed to decompose the noisy image into *D* = 5 levels. The parameters corresponding to the other comparative methods were used as specified by authors for best performance. The window size for performing the GoF test in the proposed methods was selected to be 5 × 5, though experiments with other window sizes including 3 × 3, 7 × 7 yielded similar results.

Table 1 presents the PSNR values obtained by applying various denoising methods on the selected test images. These PSNR values represent the average values taken over twenty iterations. The highest PSNR value is highlighted in *shaded bold*, while the second highest PSNR value is highlighted in *bold* (without shade) to underline the two best performing denoising algorithm at each noise level. The results in Table 1 demonstrate the superior performance of the proposed *GoFShrink-DT* against the selected state of the art of image denoising at all the noise levels for all the test images. Note that the *GoFShrink-TI* showed competitive performance when with other comparative image denoising methods for natural as well as medical images.

**Table 1:** Comparison of the proposed methods with the state-of-the-art image denoising methods in terms of output PSNR for a range of input noise levels *σ* = 10 to *σ* = 50.

*σ*	10	20	30	40	50	10	20	30	40	50
Input PSNR	28.13	22.11	18.59	16	14.15	28.13	22.11	18.59	16	14.15
**Input Image**	**Lena** (512 × 512)					**Barbara** (512 × 512)				
**BiShrink** [33]	34.31	30.97	29.12	27.67	26.55	32.93	28.82	26.64	25.09	23.99
**iTVD** [27]	33.63	30.82	29.25	28.19	**27.37**	28.79	25.72	24.55	23.91	23.47
**aTVD** [27]	32.90	30.19	28.70	27.72	26.95	27.77	25.10	24.14	23.60	23.23
**BeltDen** [28]	34.15	31.12	29.32	27.41	27.32	30.15	26.57	25.01	24.01	23.56
**PaPCA** [24]	34.27	31.40	29.67	28.09	26.75	32.57	29.29	26.97	25.39	24.17
**Surelet** [12]	34.37	30.92	29.10	27.75	26.89	32.47	28.21	26.01	24.66	23.78
**NeighSure** [18]	34.61	31.36	29.68	28.30	27.31	33.32	29.27	27.18	25.84	24.79
**cSM-EB** [15]	34.09	30.95	29.18	27.97	27.05	32.53	28.57	26.46	25.15	24.27
**GoFShrink-TI**	**34.67**	**31.46**	**29.69**	**28.29**	27.28	**33.39**	**29.47**	**27.31**	**26.08**	**25.01**
**GoFShrink-DT**	**34.72**	**31.74**	**29.97**	**28.67**	**27.68**	**33.78**	**30.06**	**27.89**	**26.29**	**25.10**
**Input Image**	**Peppers** (512 × 512)					**View** (512 × 512)				
**BiShrink**	32.51	28.66	26.65	25.22	24.15	33.74	30.27	28.61	27.67	26.74
**iTVD**	33.22	30.98	29.40	**28.35**	**27.37**	33.21	30.60	29.30	**28.44**	27.79
**aTVD**	32.74	30.54	29.20	28.20	27.14	32.57	30.14	28.95	28.17	**27.58**
**BeltDen**	**33.48**	**31.06**	29.47	27.52	27.01	33.86	30.91	**29.32**	27.49	27.51
**PaPCA**	33.24	30.95	**29.46**	28.00	26.74	**34.17**	**30.97**	29.19	27.65	26.32
**Surelet**	32.57	28.51	26.12	24.65	23.61	33.51	30.27	28.52	27.64	26.92
**NeighSure**	**33.31**	30.62	29.19	28.09	27.21	34.17	30.84	29.17	28.23	27.52
**cSM-EB**	32.72	29.14	26.11	26.01	24.91	33.71	30.61	29.16	28.27	27.61
**GoFShrink-TI**	33.00	30.77	29.24	28.08	27.13	34.11	30.67	28.93	27.85	27.11
**GoFShrink-DT**	33.07	**31.06**	**29.64**	**28.56**	**27.61**	**34.45**	**31.09**	**29.51**	**28.69**	**28.11**
**Input Image**	**Aeroplane** (512 × 512)					**Cameraman** (512 × 512)				
**BiShrink**	34.29	30.64	28.57	27.18	25.97	32.09	28.18	26.03	24.67	23.71
**iTVD**	34.04	30.74	28.92	27.67	26.75	31.28	28.16	26.43	25.21	**24.41**
**aTVD**	33.28	30.10	28.36	27.17	26.30	30.53	27.57	25.88	24.77	23.95
**BeltDen**	34.52	31.07	29.09	27.10	26.88	32.07	28.37	26.40	25.20	**24.48**
**PaPCA**	34.62	**31.34**	**29.47**	27.77	26.44	**32.80**	**29.16**	**26.91**	**25.42**	24.21
**Surelet**	34.52	30.89	28.91	27.55	26.53	31.97	28.03	26.09	24.65	23.71
**NieghSure**	34.65	31.13	29.06	27.72	26.68	**32.62**	28.51	26.37	25.00	24.01
**cSM-EB**	34.01	30.51	28.63	27.35	26.43	32.04	28.14	26.11	24.76	23.81
**GoFShrink-TI**	**34.87**	31.31	29.28	**27.87**	**26.77**	32.33	28.35	26.36	25.07	24.11
**GoFShrink-DT**	**35.23**	**31.72**	**29.70**	**28.29**	**27.26**	32.46	**28.57**	**26.71**	**25.34**	24.36
**Input Image**	**Medical Side MRI Image** (256 × 256)					**Multi-focus Image** (256 × 256)				
**BiShrink**	34.32	30.33	28.12	26.51	25.31	37.06	33.24	31.17	29.61	28.33
**iTVD**	32.91	29.42	27.61	26.37	25.40	37.27	34.02	**32.18**	**30.88**	29.86
**aTVD**	31.95	28.61	26.87	25.68	24.77	36.86	33.68	31.90	30.66	**29.69**
**BeltDen**	33.51	29.93	27.93	26.26	25.36	37.47	33.97	31.64	28.84	28.79
**PaPCA**	34.45	31.14	**29.14**	**27.48**	26.04	36.28	33.44	31.65	29.74	28.27
**Surelet**	34.51	30.44	28.23	26.72	25.56	36.84	32.99	30.67	29.25	28.15
**NieghSure**	34.91	30.69	28.49	26.98	25.73	37.64	34.01	32.11	30.60	29.66
**cSM-EB**	33.92	30.18	28.09	26.75	25.61	37.14	33.68	31.69	30.35	29.37
**GoFShrink-TI**	**35.04**	**31.17**	28.85	27.28	**26.12**	**37.81**	**34.03**	31.92	30.32	29.09
**GoFShrink-DT**	**35.41**	**31.46**	**29.20**	**27.72**	**26.56**	**38.07**	**34.23**	**32.23**	**30.91**	**29.89**

For the input image *Barbara* (of size 512 × 512), the *GoFShrink-DT* and the *GoFShrink-TI* outperformed other denoising methods at all noise levels. The best results were shown by the *GoFShrink-DT* which beat the rest of the denoising methods including the second best *GoFShrink-TI* method by a considerable margin. The *GoFShrink-DT* also demonstrated superior performance for *Lena* image (of size 512 × 512) at all noise levels while the second best results were shown by *GoFShrink-TI* at noise levels 10 ≤ *σ* ≤ 40 and *iTVD* at *σ* = 50, which outperformed *GoFShrink-TI* by a small margin.

For *Aeroplane* and *Side MRI* images, the proposed *GoFShrink-DT* outperformed all the comparative methods at all noise levels, while second best results were obtained by *GoFShrink-TI* and *PaPCA* alternatively at different noise levels. The second best performance was demonstrated by the *GoFShrink-TI* for *Aeroplane* image at noise level *σ* = 10, 40 & 50, while the *PaPCA* yielded second best results for *σ* = 20 & 30. Similarly, for *Brain MRI* image, the *GoFShrink-TI* offered second best performance at input noise levels *σ* = 10, 20 & 50 while *PaPCA* yielded second highest PSNR values for *σ* = 30 & 40.

For *Peppers* image (of size 512 × 512) at *σ* = 10 & 20, the *BeltDen* yielded best performance in terms of output PSNRs followed by the *NeighSure* at *σ* = 10 and the *GoFShrink-DT* at *σ* = 20. For noise levels *σ* ≥ 30 *GoFShrink-DT* yielded best results.

For *Cameraman* image (of size 256 × 256), the *PaPCA* method demonstrated best performance against the rest of the denoising methods for 10 ≤ *σ* ≤ 40. However, at *σ* = 50, *BeltDen* yields the best results. The *GoFShrink-DT* shows the second best performance for *Cameraman* image at 20 ≤ *σ* ≤ 40. The *NeighSure* exhibited second best performance at the noise level *σ* = 10, while at noise level *σ* = 50, *iTVD* yielded second highest PSNR values. Even though, the *GoFShrink-TI* failed to be among top two performing methods for *Cameraman* image, it showed competitive performance against the best methods.

Similarly, the *GoFShrink-DT* outperformed the comparative state of the art methods for *View* and *Multi-focus* images (of size 512 × 512) at all noise levels. For *Multi-focus* image, the *GoFShrink-TI* yielded next best results at noise level *σ* ≤ 20, while the *iTVD* showed second best performance at *σ* = 30 & 40. For the *View* image, the *PaPCA* yielded second best results at *σ* ≤ 20, while the *BeltDen*, *iTVD* and *aTVD* were second best respectively for noise levels *σ* = 30, 40 & 50.

Table 2 presents the qualitative analysis of the denoised images obtained from the comparative state of the art methods along with the proposed *GoFShrink-DT* method. For that purpose, we obtain results for input images ‘Lena’, ‘Plane’, ‘Peppers’ and ‘MRI’. It can be observed that the denoised images obtained from the proposed method yields highest SSIM and FSIM values on most occasions. In cases where other methods yield better results, the proposed method still remains quite competitive. Among the state of the art, *PaPCA* and *BeltDen* yields the best results in terms of the SSIM and FSIM values.

**Table 2:** Comparison of the proposed methods with the state-of-the-art image denoising methods in terms of structural similarity (SSIM) and feature similarity (FSIM) for a range of input noise levels *σ* = 10 to *σ* = 50.

*σ*		10	20	30	40	50	10	20	30	40	50
Input PSNR		28.13	22.11	18.59	16	14.15	28.13	22.11	18.59	16	14.15
**Input Image**		**Lena** (512 × 512)					**Pane** (512 × 512)				
**Input**	SSIM	0.436	0.251	0.167	0.119	0.089	0.396	0.253	0.181	0.137	0.106
	FSIM	0.952	0.866	0.783	0.715	0.656	0.954	0.873	0.799	0.736	0.682
**BiShrink**	SSIM	0.586	0.485	0.421	0.374	0.336	0.546	0.443	0.381	0.332	0.300
	FSIM	0.976	0.946	0.917	0.891	0.867	0.973	0.937	0.905	0.875	0.847
**iTVD**	SSIM	0.551	0.456	0.401	0.359	0.329	0.525	0.435	0.378	0.339	0.305
	FSIM	0.969	0.944	0.925	0.908	0.894	0.966	0.938	0.916	0.898	0.882
**aTVD**	SSIM	0.518	0.426	0.374	0.335	0.307	0.493	0.406	0.352	0.316	0.286
	FSIM	0.962	0.934	0.913	0.894	0.879	0.958	0.925	0.901	0.883	0.867
**BeltDen**	SSIM	0.599	0.490	0.420	0.352	0.334	0.572	**0.460**	0.392	0.331	0.314
	FSIM	0.977	0.954	0.931	0.891	0.896	0.974	0.949	0.924	0.886	0.885
**PaPCA**	SSIM	0.611	0.493	0.433	0.399	0.335	0.577	0.455	0.398	0.360	0.310
	FSIM	0.976	0.956	0.932	**0.926**	0.873	0.976	**0.951**	0.924	**0.911**	0.864
**Surelet**	SSIM	0.614	0.496	0.430	0.384	0.348	0.572	0.455	0.390	0.346	0.312
	FSIM	0.976	0.944	0.916	0.892	0.873	0.973	0.937	0.907	0.881	0.860
**NeighSure**	SSIM	0.608	0.502	0.436	0.392	0.357	**0.559**	0.452	0.388	0.346	0.313
	FSIM	0.979	0.954	0.931	0.909	0.889	0.975	0.941	0.912	0.888	0.865
**cSM-EB**	SSIM	0.610	0.497	0.431	0.386	0.352	0.551	0.443	0.381	0.338	0.307
	FSIM	0.977	0.950	0.926	0.904	0.886	0.972	0.937	0.907	0.881	0.861
**GoFShrink-DT**	SSIM	0.599	**0.509**	**0.452**	**0.410**	**0.376**	0.557	0.457	**0.400**	**0.356**	**0.324**
	FSIM	**0.979**	**0.957**	**0.937**	0.921	**0.906**	**0.977**	0.949	**0.925**	0.905	**0.888**
**Input Image**		**Peppers** (512 × 512)					**MRI** (512 × 512)				
**Input**	SSIM	0.483	0.263	0.170	0.119	0.088	0.519	0.337	0.239	0.180	0.141
	FSIM	0.952	0.865	0.784	0.713	0.655	0.886	0.761	0.665	0.594	0.538
**BiShrink**	SSIM	0.492	0.418	0.371	0.338	0.313	0.664	0.558	0.477	0.417	0.373
	FSIM	0.972	0.940	0.909	0.884	0.860	0.944	0.895	0.857	0.829	0.805
**iTVD**	SSIM	0.507	0.425	0.378	0.348	0.323	0.618	0.505	0.433	0.381	0.347
	FSIM	0.974	0.952	0.931	0.915	0.901	0.922	0.866	0.826	0.797	0.777
**aTVD**	SSIM	0.477	0.400	0.358	0.330	0.306	0.586	0.465	0.393	0.342	0.308
	FSIM	0.970	0.946	0.929	0.914	0.900	0.904	0.840	0.797	0.767	0.745
**BeltDen**	SSIM	0.549	0.457	0.400	0.343	0.329	0.646	0.545	0.478	0.430	0.363
	FSIM	0.975	0.951	0.933	0.907	0.893	0.937	0.890	0.858	0.829	0.789
**PaPCA**	SSIM	0.587	**0.471**	**0.410**	**0.378**	0.325	0.668	0.563	0.493	0.425	0.393
	FSIM	0.973	0.953	0.929	0.925	0.876	0.956	0.915	**0.889**	**0.854**	**0.848**
**Surelet**	SSIM	**0.597**	0.458	0.396	0.359	0.330	0.670	0.569	0.494	0.439	0.394
	FSIM	0.974	0.942	0.915	0.891	0.875	0.945	0.896	0.859	0.832	0.810
**NeighSure**	SSIM	0.578	0.432	0.384	0.351	0.324	0.672	0.565	0.492	0.432	0.390
	FSIM	0.975	0.946	0.921	0.901	0.880	0.950	0.902	0.865	0.836	0.815
**cSM-EB**	SSIM	0.591	0.460	0.398	0.361	0.331	0.653	0.549	0.479	0.427	0.387
	FSIM	0.973	0.946	0.920	0.901	0.882	0.942	0.896	0.860	0.836	0.815
**GoFShrink-DT**	SSIM	0.514	0.435	0.393	0.362	**0.338**	**0.687**	**0.586**	**0.506**	**0.442**	**0.393**
	FSIM	**0.977**	**0.954**	**0.935**	**0.919**	**0.903**	**0.956**	**0.917**	0.874	0.841	0.818

The above results and discussion clearly demonstrate the efficiency of the GOF based methods against the state of the art denoising methods for a variety of practical input images. Similarly, the *GoFShrink-TI* also showed competitive performance against the state of the art in image denoising. From the state of the art methods, *PaPCA* and *iTVD* yielded good performance against the proposed methods while the *NeighSure* and the *Surelet* have also been competitive.

**Fig 8:**
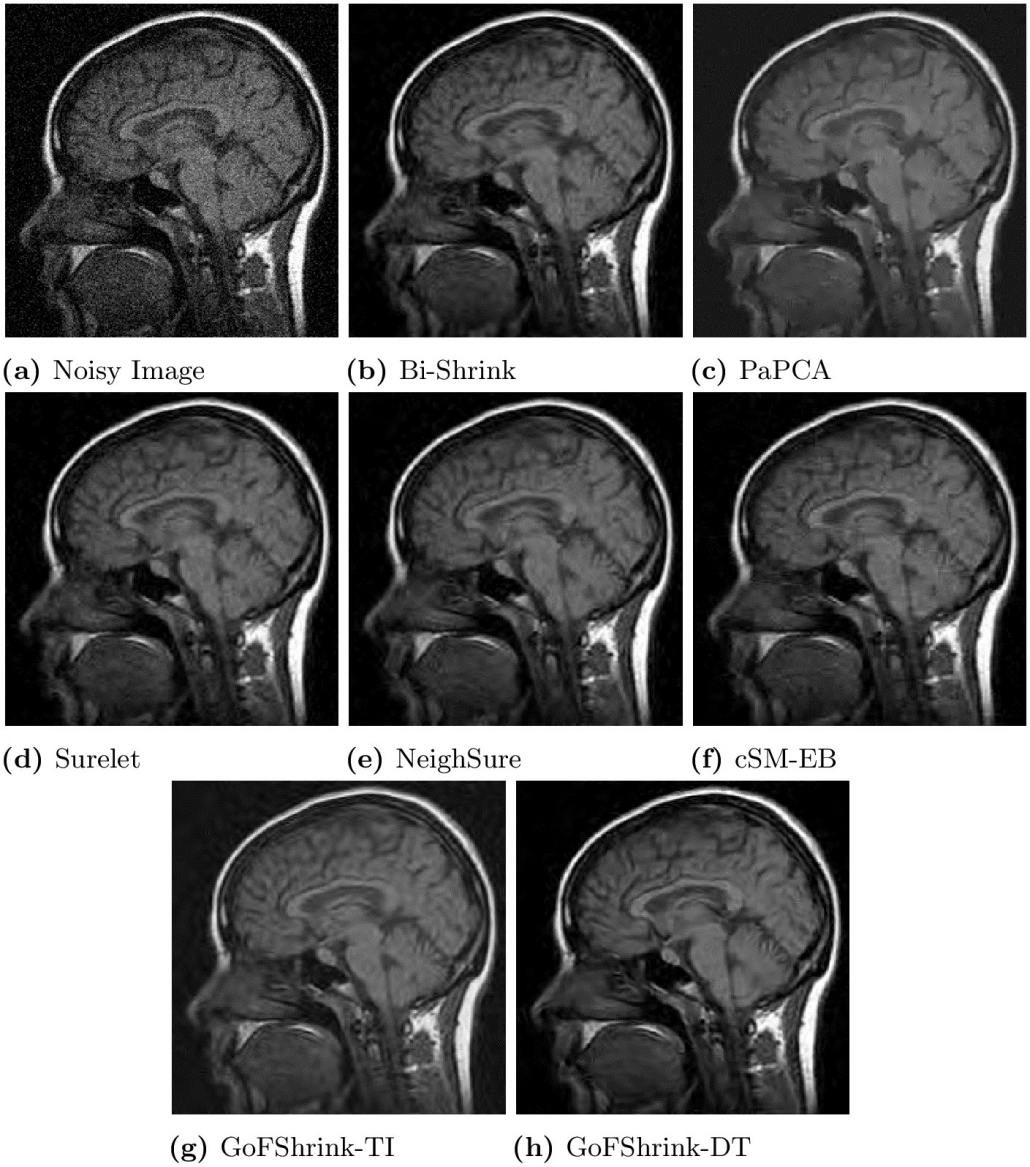
Visual results for several state-of-the-art image denoising methods on the *Side MRI image* of a brain corrupted with the noise level *σ* = 20. This figure is composed of (a) noisy image; (b) denoised image from *Bi-Shrink*; (c) *PaPCA*; (d) *Surelet*; (e) *NieghSure*; (f) *cSM-EB*; (g) *GoFShrink-TI*; and (h) *GoFShrink-DT*.

To show the visual quality of the recovered images by various denoising methods, we take a specific case of a *Brain MRI* image in Fig 8, corrupted with WGN at *σ* = 20. The Fig 8(a) shows noisy versions of the *Brain MRI* image while Fig 8(b)–8(h) show the corresponding denoised images obtained by employing *BiShrink*, *PaPCA*, *Surelet*, *NeighSure*, *cSM-EB*, *GoFShrink-TI* and *GoFShrink-DT*, respectively. It can be noticed that the *GoFShrink-DT* retained the image details and avoided artifacts thereby providing the best visual quality denoised image as compared to the other denoising methods. The *GoFShrink-TI* though contains some artifacts but it also manages to preserve important details as compared to *NeighSure*, *Surelet* and *BiShrink* which also yielded artifacts. The *cSM-EB* performed comparatively better but fails to capture the clarity as evident in *GoFShrink-DT* results. The *PaPCA* demonstrated visually pleasing results with lesser artifacts, however, the denoised image is over-smoothed and it is hard to differentiate between smoother regions and inherent image discontinuities. We also computed the difference images corresponding to all the denoised images and then estimated the power of the difference images. It was observed that least power of the difference image was yielded by proposed methods i.e. 38.7 & 50.9 while the comparative methods yielded higher power difference images.

**Fig 9:**
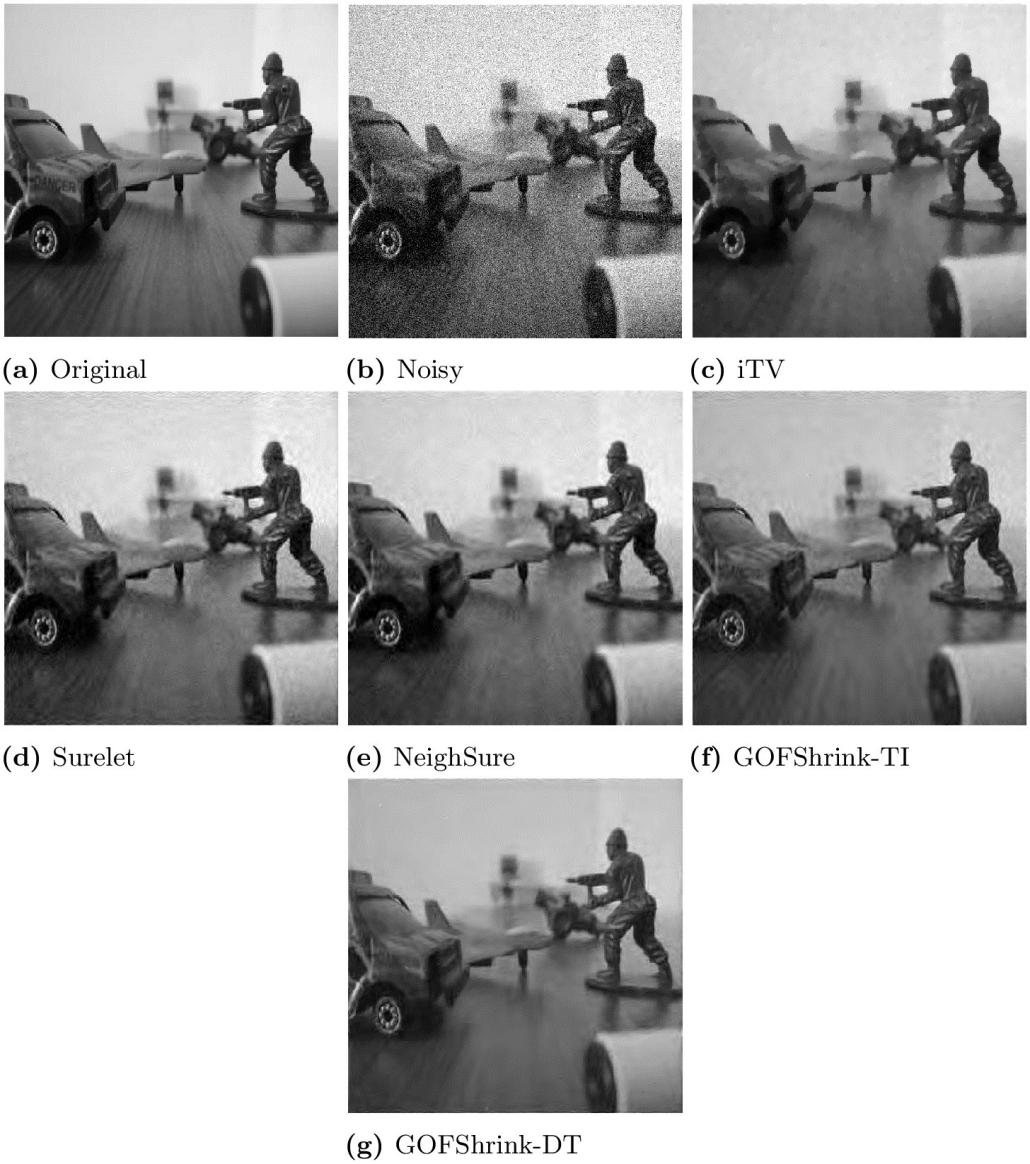
Results of several state of the art image denoising methods on *Multi-focus* image corrupted with noise with standard deviation *σ* = 30; (a) original image (b) noisy image (c) denoised image by *iTVD* and a zoomed in region (d) denoised image by *Surelet* and a zoomed in region (e) denoised image by *NeighSure* and a zoomed in region (f) denoised image by *GoFShrink-TI* and a zoomed in region (g) denoised image by *GoFShrink-DT* and a zoomed in region.

In Fig 9, the performance of the proposed *GoFShrink-TI* and the *GoFShrink-DT* is compared with the *iTVD*, *Surelet* and *NeighSure* for the *Multi-focus* image. It can be observed that the denoised image obtained through the proposed *GoFShrink-DT* bears striking resemblance to the original image as it contains least artifacts and recovers all of the important details when compared to the other methods. Second best results were shown by the *GoFShrink-TI* which recovered all the details with few artifacts, see Fig 9(f). The *NeighSure* and the *Surelet* yielded more artifacts in Fig 9(d) & 9(e) even though image details were preserved. Contrarily, the *iTVD* over-smoothed the detailed regions leading to a poor estimate of the original image as shown in Fig 9(c). Another evidence of the best visual performance by the proposed methods is the least power of difference images (obtained by subtracting denoised images from original) 38.18 and 43.82 respectively while the comparative methods *Surelet* and *NeighSure* yield 45.64 and 54.71 respectively. Even though the *iTVD* yields lower noise power compared to the *GoFShrink-TI*, the visual quality of its denoised image is not particularly impressive.

**Fig 10:**
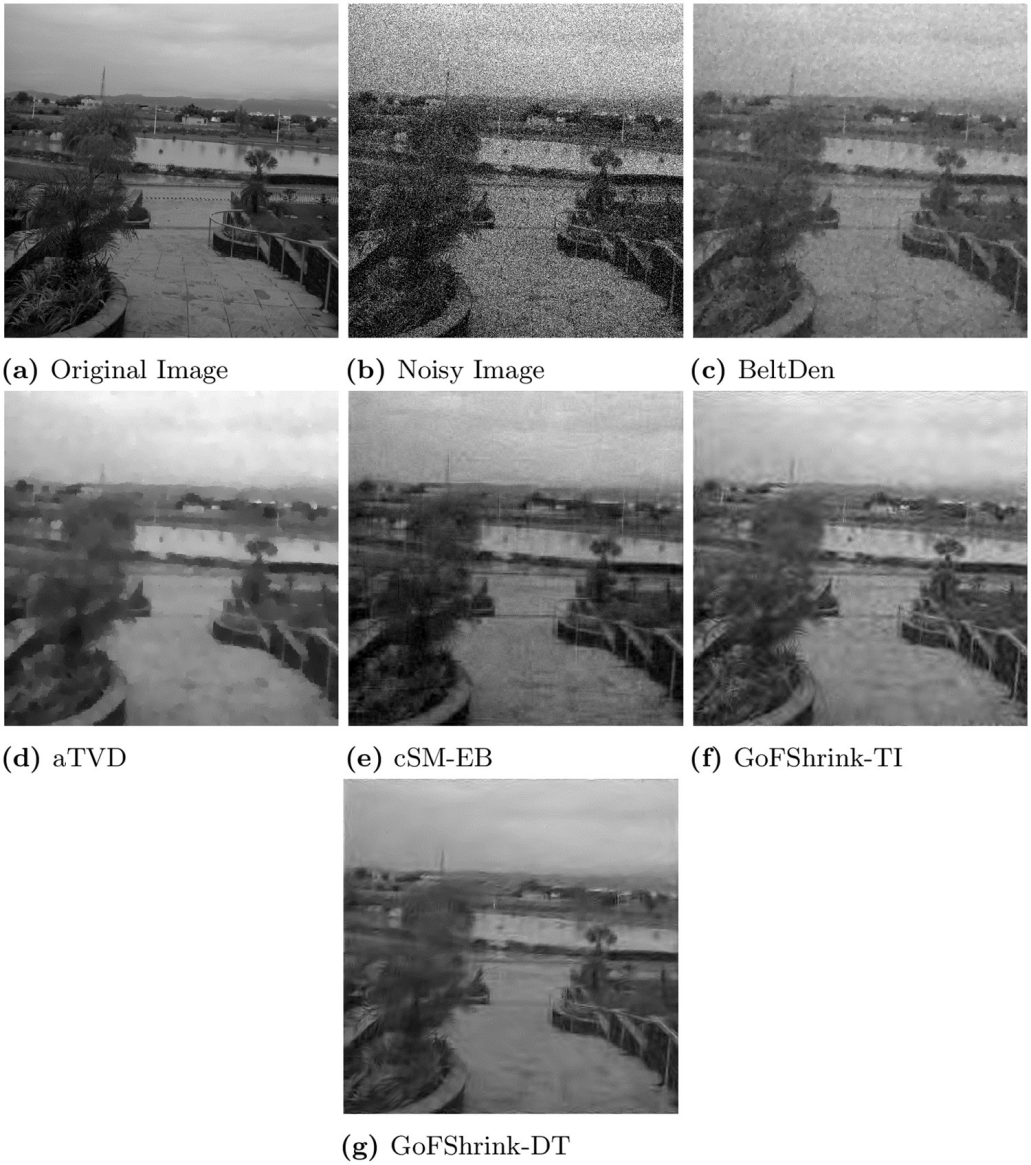
Visual performance comparison of various denoising methods on the *View* image at higher noise level *σ* = 40. This figure is composed of (a) original image; (b) noisy image and denoised images from (d) *aTVD*; (e) *cSM-EB*; (f) *GoFShrink-TI*; and (g) *GoFShrink-DT*.

In Fig 10, shows the actual and noisy view image along with the denoised images obtained from the *BeltDen*, *aTVD* and cSM-EB and the proposed for the input noise level *σ* = 40. Note that the denoised image obtained from *GoFShrink-DT* in Fig 10(g) yielded few artifacts with most details intact. The *GoFShrink-TI* also managed to recover important details when compared against the state of the art methods but it also yielded considerable amount of artifacts. The denoised images from other comparative methods including the *BeltDen* and the cSM-EB show significant artifacts. The aTVD yielded lesser artifacts as compared to *BeltDen*, cSM-E, albeit few line artifacts are still present while image details are missing.

In order to validate our work, the proposed *GoFShrink-DT* is also compared against the *NLM* method, which is a computationally intensive state of the art method known for its effective denoising performance. For this purpose, *Brain MRI* and *Multi-focus* images have been used. The denoised images obtained from the the *NLM* and the *GoFShrink-DT*, at input noise level *σ* = 20 & 30 (i.e. noisy MRI image with PSNR = 22.11 & 18.59), have been displayed in S2 Fig which is provided as supplementary material with this work. S2 Fig also reports the corresponding PSNR values of the noisy and the denoised images. The first column of the *Auxiliary Fig* 2, shows noisy images while the second and third columns show denoised images obtained from the *NLM* and the *GoFShrink-DT* respectively. It is evident that *NLM* method yielded higher PSNRs and also managed to smooth out noise very effectively. However, *NLM* smooths images discontinuities or edges thereby loosing important details of the MRI image. Contrarily, the *GoFShrink* yielded comparatively less PSNR but it recovered important signal details which might be useful in the clinical diagnosis.

Similar trends can be observed in the bottom two rows of the *Auxiliary Fig* 2 where the *NLM* over smooths the *Multi-focus* image at input noise level *σ* = 20 & 30 while yielding comparatively higher PSNR values than those of the proposed method. However, the proposed *GoFShrink* gives sharper denoised image with more signal details.

## 6 Conclusion

A class of multiscale image denoising algorithms have been proposed which employ the goodness of fit test on multiple image scales obtained from discrete wavelet transform (DWT) and dual tree complex wavelet transform (DT-CWT). The Anderson Darling (AD) statistics have been employed, within the framework of GoF test, on the wavelet coefficients of the noisy image to compute the distance between the empirical distribution function (EDF) of local coefficients and the CDF of reference Gaussian noise. A local thresholding function is then used to classify the wavelet coefficients as belonging to signal or noise depending on the given probability of false alarm (*P*_*fa*_) and the estimated AD statistic. The signal coefficients are retained while the noise coefficients are discarded to yield the denoised image. While the current work only deals with the case of Gaussian noise, the proposed scheme has potential to remove any type of noise with prior knowledge of the noise distribution. The proposed methods have been shown to outperform the state-of-the-art image denoising methods on a variety of input images ranging from standard test datasets to medical and diffusion images. The results have revealed that from the two proposed methods, the *GoFShrink-DT* (based on DT-CWT) has outperformed the *GoFShrink-TI* (based on DWT) which was expected given directional selectivity and translation invariance of the DT-CWT transform.

## Supporting information

S1 Fig Standard Input test images (a) Lena (b) Barbara (c) Peppers (d) Plane (e) Cameraman (g) Brain MRI. (TIF)

S2 Fig Comparison of the denoising performance of the proposed *GoFShrink-DT* against the *NLM* method on *Multifocus* and *MRI* datasets, whereby first column displays the noisy input images (at *σ* = 20 & 30) while second and third columns show denoised images by the *NLM* and the *GoFShrink-DT* respectively. In addition, PSNR values of each image have also been reported.
(TIF)
