# Supplementary figures and images for: Multiscale image denoising using goodness-of-fit test based on EDF statistics
Source: PLoS One. 2019 May 10;14(5):e0216197. doi: 10.1371/journal.pone.0216197 (PMC6510407; doi:10.1371/journal.pone.0216197)

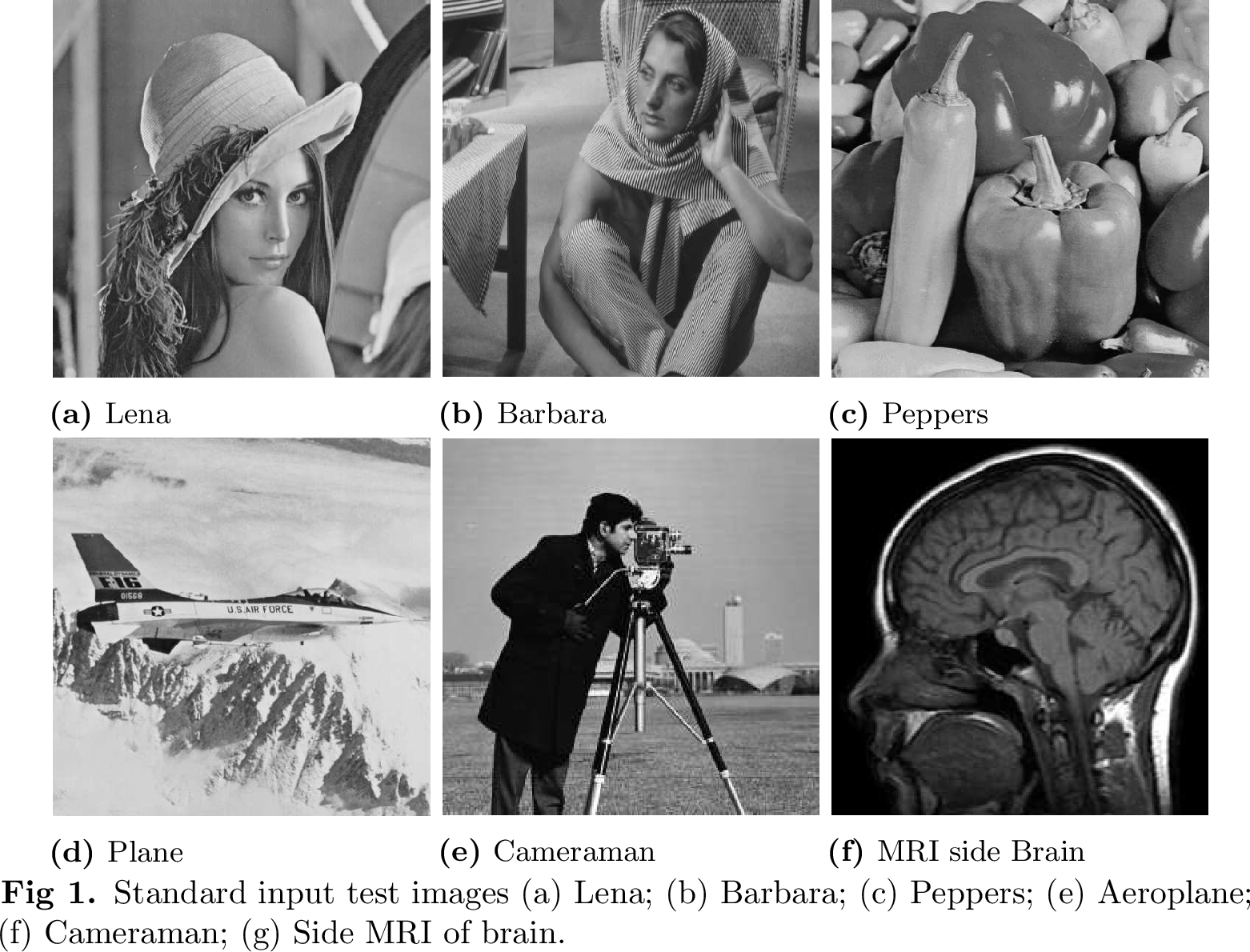

Supplement: S1 Fig — (TIF) [file pone.0216197.s001.tif]

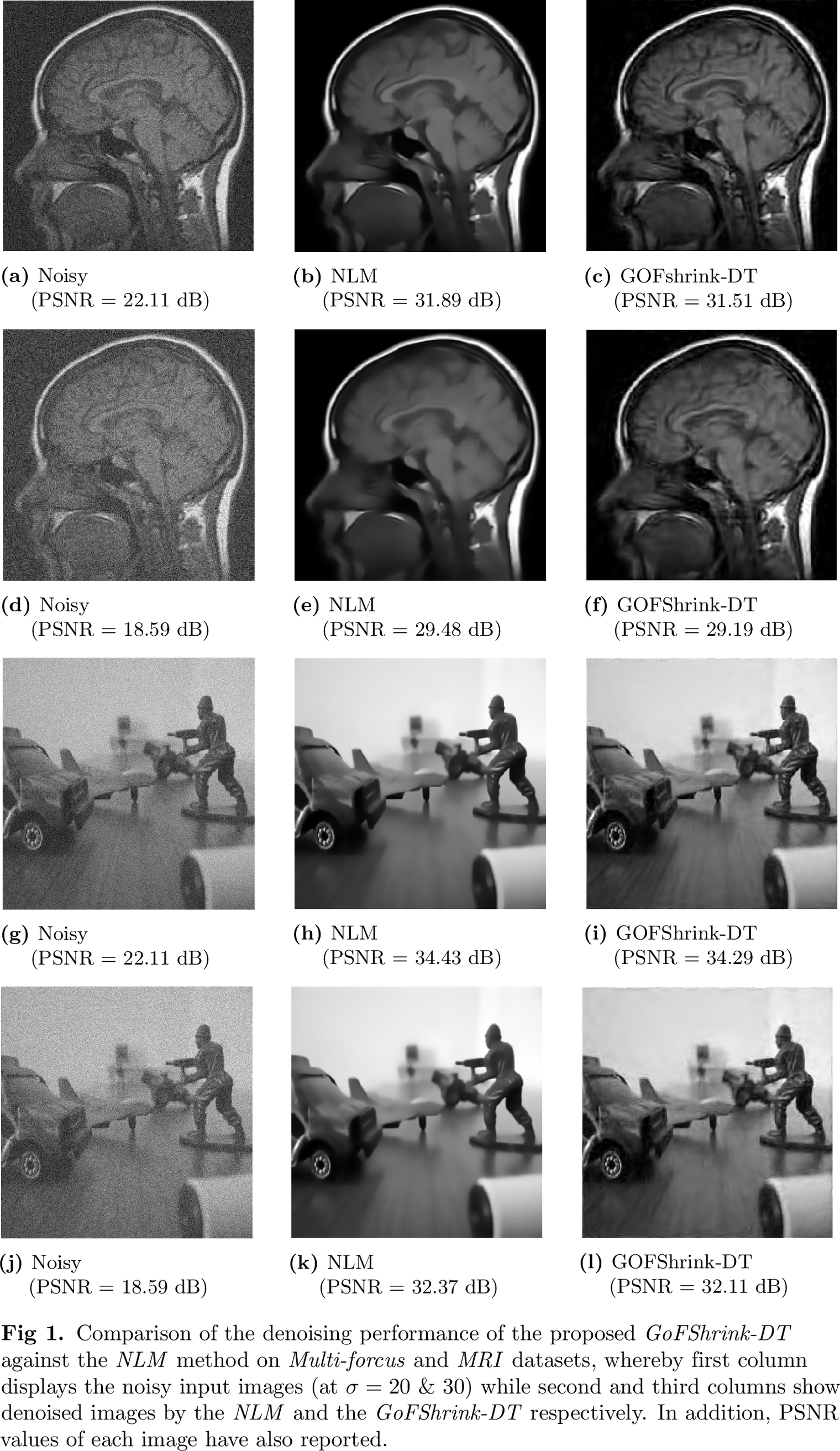

Supplement: S2 Fig — In addition, PSNR values of each image have also been reported. (TIF) [file pone.0216197.s002.tif]
